# The discriminative capacity of known predictors of outcome for patients with acute exacerbations of copd treated in the ICU

**DOI:** 10.1186/2197-425X-3-S1-A451

**Published:** 2015-10-01

**Authors:** D Ramnarain, E Brands

**Affiliations:** Elisabeth Tweesteden Hospital Tilburg, Intensive Care Medicine, Tilburg, the Netherlands; Elisabeth Tweesteden Hospital Tilburg, Tilburg, the Netherlands

## Introduction

Patients with acute exacerbations of chronic obstructive pulmonary disease (AECOPD) visiting the EDs are often hospitalized. Assessing the severity of AECOPD is mandatory to guide decisions of orientation as well as intensity of monitoring treatment and follow-up during and after the acute episode. Despite several published scores for predicting outcome in AECOPD a simple and clinical prediction model is still lacking for patients admitted to the ICU.

## Objectives

In our ICU the past years standard mortality rate of AECOPD exceeded the national standard. Therefore we studied this cohort of patients from January 2011 until March 2014 retrospectively aiming to assess the validity of several known predictive scoring models ( e.g. BAP-65 score, DECAF-score, eMRCD score and CAOS- score) and to find possible factors that could also be associated with poor outcome of these patients treated in the ICU [1].

## Methods

All data from patients admitted to the ICU form January 2011 until March 2014 were studied using medical files and Mediscore, a national database of all patients admitted in ICUs in the Netherlands. To evaluate difference in survivors and non-survivors of AECOPD we used chi square test and non-parametric tests. Area under the receiver operating characteristic-ROC analysis was used to assess the discriminative capacity of several prediction models.

## Results

There were 163 patients with mean Apache 4 score 57.2 (16.6), Apache 2 score 17.4(5.1). N = 115 survivors and n = 48 non-survivors. Mean age 67, sd9.3. BMI 24.5 (sd 5.1). 58% were classified as GOLD IV and 37% used long-term oxygen therapy. 50% of patients were limited by the disease. 20% were “bed-chair” mobilisers and 30% was housebound. Coronary disease was a known co-morbidity in 22.7% with heart failure in 11%. 24.5% of patients was diagnosed with pneumonia at admission.

We found no significant difference between survivors and non-survivors in demographic data, severity of COPD, mobility or co-morbidities. Only CAOS score and Apache 4 were significant different in both groups with a poor discriminative value in predicting in hospital- and 180-day mortality (AUROC 0.644 and 0.652 resp.). See Table 1:

**Table 1:** Table 1

Prognostic variable	In hospital mortality (AUROC)	180-day mortality (AUROC)	Significance
CAOS	0.644	0.580	n.s.
Apache 4	0.652	0.577	n.s.
BAP-65	0.543	0.477	n.s.
DECAF	0.523	0.522	n.s.
eMRCD	0.555	0.563	n.s.

## Conclusions

In patients with AECOPD admitted to our ICU for further treatment the known prediction models have poor discriminative capacity in predicting in hospital as well as 180- day mortality. Further research to find predictive scores for AECOPD is mandatory.
